# Activation of PPARγ Inhibits TLR4 Signal Transduction Pathway in Melanoma Cancer *In Vitro*

**DOI:** 10.34172/apb.2020.056

**Published:** 2020-05-11

**Authors:** Nasim Dana, Golnaz Vaseghi, Shaghayegh Haghjooy Javanmard

**Affiliations:** ^1^Applied Physiology Research Center, Cardiovascular Research Institute, Isfahan University of Medical sciences, Isfahan, Iran.; ^2^Isfahan Cardiovascular Research Center, Cardiovascular Research Institute, Isfahan University of Medical sciences, Isfahan, Iran.

**Keywords:** Peroxisome proliferatoractivated receptor, Toll-like receptor 4, Melanoma, Pioglitazone

## Abstract

***Purpose:*** Although peroxisome proliferator-activated receptor γ (PPARγ) is known as a regulator of fatty acid storage, fat cell differentiation, glucose and lipid metabolism, recent studies show that PPARγ has anticancer effects. The mechanisms of PPARγ activation in melanoma cancer remain unclarified. Recently, increased TLR4 expression has been associated with the melanoma cancer progression. We investigated whether the anti-cancer effect of PPARγ is through regulating TLR4 signaling pathway.

***Methods:*** Mouse melanoma cells (B16F10) were treated in different groups: control, pioglitazone (1, 10, 100, 300 µmol/L), lipopolysaccharide (LPS) (5 µg/mL) and LPS + pioglitazone. In another experiment, they were treated with CLI-095 (1 μM), and after 1 hour pioglitazone was added and subsequently stimulated with LPS. MTT assay was performed to measure the cell viability *in vitro*. The expression of *Tlr4, Myd88, Nf-κb* genes were evaluated by quantitative reverse transcription PCR (qRT-PCR) in different groups. The concentration of tumor necrosis factor alpha and Interleukin 1 beta in the cell culture medium were measured by enzyme-linked immunosorbent assay (ELISA) kits.

***Results:*** We show that activation of PPARγ by its agonist, pioglitazone, reduces cell proliferation, *Tlr-4*, *Myd-88*, *Nf-kb* mRNA expression, and tumor necrosis factor-alpha (TNF-α) production but not interleukin-1 β (IL-1β) in B16F10 LPS–stimulated cells *in vitro*. Moreover, treatment of B16F10 cells with TLR4 inhibitor prior treatment with pioglitazone indicate that the anticancer effects of pioglitazone on melanoma cells was dependent on TLR4.

***Conclusion:*** The results indicate that pioglitazone has a beneficial protective effect against melanoma by affecting the TLR4 signaling pathway.

## Introduction

Melanoma is a type of skin cancer that begins in the melanocytes after malignant transformation such as genetic mutations and tumor microenvironmental alterations in these cells.^1^ Some of these changes are mediated by dysregulation of the NF-κB (nuclear factor kappa-light-chain-enhancer of activated B cells). NF-κB is a major anti-apoptotic factor and has a fundamental role in melanoma progression.^2,3^

Peroxisome proliferator-activated receptor gamma (PPARγ) is a transcription factor that belongs to the superfamily of nuclear receptors.^4^ PPARγ can regulate gene expression of proinflammatory genes such as NF-κB. Pioglitazone is known to be a ligand for PPARγ.^5^ It has been shown that activation of PPARγ can inhibit the proliferation of melanoma cells but its mechanism is not clear.

Melanoma tumors by using NF-κB can achieve to survival, proliferation and resistance to apoptosis.^6^

One of the reasons for the increase in NF-κB levels in melanoma cells can be over-expression of toll like receptor 4 by melanoma cells.^7^ Toll-like receptors (TLRs), like other pattern recognition receptors, are responsible for recognizing different molecular patterns for example molecular patterns of pathogens.^8^ It has been shown that TLR4 is overexpressed in different cancers. It plays different roles in cancer development and progression.^9^

TLR4 signaling in response to lipopolysaccharide (LPS) stimulation, increase migration, invasion and adhesive properties of tumor cells such as breast, esophageal and colorectal cancer cell lines.^10-13^

Recent evidence suggests that PPARs and TLRs signaling pathways have crosstalk in different diseases.^14^ However, there is a little knowledge about the interaction between TLR4 pathway and the anti-inflammatory effect of PPARγ in cancer. So the aim of this study was to investigate the probable relation between PPARγ and TLR4 in melanoma cancer *in vitro*.

## Materials and Methods

### Cell and reagents

The mouse melanoma cell line (B16F10) purchased from the National Cell Bank of Iran (affiliated to Pasteur Institute, Tehran, Iran). Culture media and supplements were obtained from Gibco BRL (Carlsbad, CA, USA. Ultrapure LPS-EB from *E. coli 0111: B4* and CLI-095 [resatorvid, ethyl (6*R* )-6-[*N* -(2-chloro-4-fluorophenyl)sulfamoyl] cyclohex-1-ene-1-carboxylate] were provided by Invivogen (San Diego, CA). Pioglitazone and MTT (3-(4,5-dimethylthiazol-2-yl)-2,5-diphenyltetrazolium bromide) were produced by Sigma (St. Louis, MO, USA). ELISA kits were prepared from eBioscience (San Diego, CA, USA).

### Cell culture and treatment 

The B16-F10 cells were grown in complete media under standard conditions. Cells were seeded into the wells of the plate at densities of 10 000 cell/well (in 96 well) and 200 000 cell/well (in 24 well) for different assays and incubated for 24 hours.

After the incubation period, cells were divided into control, pioglitazone (1, 10, 100, 300 µmol/L), LPS (5 µg/mL) and LPS + pioglitazone (corresponding LPS group plus (1, 10, 100, 300 µmol/L) pioglitazone) groups. There were two different control groups in this study, one control group didn’t receive any treatment and all groups that have not treated with LPS were compared with this control. The second control group was the cells that were treated with LPS and all LPS treated groups were compared with this control. For the experiments initially, the cells were treated with different dose of pioglitazone for one hour. Following stimulation, the cells were treated with LPS subsequently. After 24 hours, MTT assay was done, mRNA levels of *Tlr-4*, *Myd-88* and *Nf-kb* were measured by real-time quantitative reverse transcription PCR (qRT-PCR). For measurable amounts of tumor necrosis factor-alpha (TNF-α) and interleukin-1 β (IL-1β) in cell culture supernatant.

### MTT assay 

B16-F10 cells were treated with or without pioglitazone and LPS as described above. Then MTT assay was done followed by 4 hours incubation of cells with MTT solution. After incubation, the medium/MTT mixtures were removed, and the formazan crystals were dissolved in DMSO. Optical densities at 570 nm were measured with a microplate reader (BioTek Instruments, Epoch, USA). The percentage cell viability was calculated by the formula according to our previous study.^15^

### RNA extraction

After treatment of B16F10 cells as described above, total RNA was extracted by using a GeneJET RNA purification kit (Thermo Scientific, (EU) Lithuania). At first RNA concentration and purity of each sample was verified and then it was converted to cDNA using a RevertAid First Strand cDNA Synthesis Kit (Fermentas, Vilnius, Lithuania).

### Quantitative reverse transcriptase polymerase chain reaction (qRT-PCR)

We used real time qRT-PCR to quantify mRNA expression levels of *Tlr - 4*, *Myd-88* and*Nf-kb*. The primer sequences for *Tlr-4*, *Myd-88*, *Nf-kb*, and beta-actin Primer sequences were as follows: (*Tlr-4*, 5′-AGTGGCTGGATTTATCCAGGTGTG-3′(fwd) and 5′-TTGAGAGGTGGTGTAAGCCATGCC-3′(rev); *Myd-88,* 5′-AAGTCTAGGAAGGCCCCAAA-3′ (fwd) and 5′-CTGGGGAGAAAACAGCTGAG-3′ (rev);*Nf-kb,* 5′- ACACGAGGCTACAACTCTGC-3′(fwd) and 5′- GGTACCCCCAGAGACCTCAT-3′(rev); *β-actin,* 5′- GCTGTATTCCCCTCCATCGTG -3′(fwd) and 5′- CACGGT TGGCCT TAGGGTTCAG -3′(rev)).

Real-time RT-PCR was performed by the Maxima SYBR Green Rox qPCR master mix kit (Fermentas, Vilnius, Lithuania). Real-time PCR reactions were performed using Corbett machine, Rotorgene 6000 (Australia). The other conditions and procedures were as described previously.^16^

The expression level of each target gene normalized with respect to the expression of housekeeping β*-actin* gene was calculated as 2^-ΔΔCt^.

All treatments were performed in triplicate wells for each condition and repeated at least twice.

### Enzyme-linked immunosorbent assay (ELISA)

B16F10 cells at a concentration of 200000 cells/well, were treated with pioglitazone (1, 10, 100, 300 µmol/L) with or without LPS (5 µg/mL). In another experiment, at first the cells were treated with CLI-095(1 µM) for one hour then pioglitazone (300 µmol/L) was added. Subsequently cells were stimulated with LPS (5 µg/mL) for 24 hours. Culture medium was collected from each well, centrifuged and stored at -80^°^C. The concentration of TNF-α and IL-1β was determined by ELISA kits (Mouse TNF-α Instant ELISA and mouse IL-1β Instant ELISA).

### Statistical analysis 

For comparison of the difference between groups unpaired Student’s t test or one-way ANOVA followed by post hoc Dunn’s multiple comparison tests was used. *P* values <0.05 were considered as statistically significant. All statistical analysis was performed using SPSS 20.

## Results

### Pioglitazone activation suppresses proliferation of B16F10 cells 

**Figure 1:**
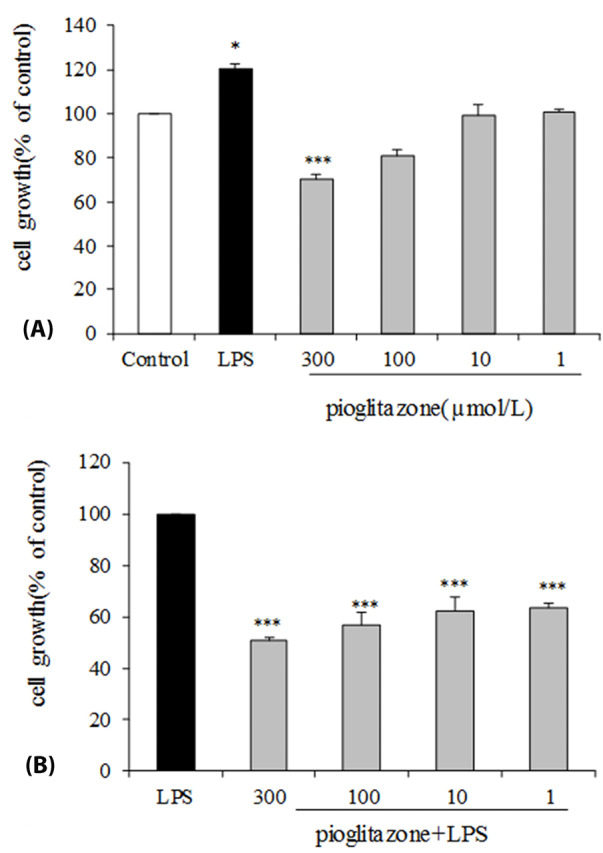
Effects of LPS-EB Ultrapure and pioglitazone treatment on B16F10 cell line viability. (A) B16F10 cells were incubated with pioglitazone (1, 10, 100, 300 µM) or LPS (5 µg/mL) for 24 h. (B) B16F10 cells were pre-incubated for 1 h with our without pioglitazone (1, 10, 100, 300 µM) then continuing the cells incubation with LPS (5 µg/mL) for 24 h. Cell viability was determined by MTT assay. * *P* <0.05; *** *P* <0.001 compared with negative control. The data are representative of three independent experiments and are expressed as mean± SEM.

The effect of pioglitazone and LPS separately on B16F10 cells proliferation was assessed. As shown in Figure 1A, treatment of B16F10 cells with LPS alone compare with control group significantly increased (*P*  < 0.05) the cell viability. Cell treatment with different concentrations of pioglitazone illustrated that only the highest concentration of pioglitazone (300 μmol/L) inhibited significantly proliferation of B16F10 cells (*P*  < 0.001). Cell stimulation with both LPS and pioglitazone indicated that pioglitazone treatment in comparison with the LPS-stimulated control group significantly inhibited the effect of LPS on cell proliferation by all of the pioglitazone doses (*P*  < 0.001; Figure 1B).

### Pioglitazone reduces expression of Tlr4, Myd-88, and Nf-kb mRNA

**Figure 2:**
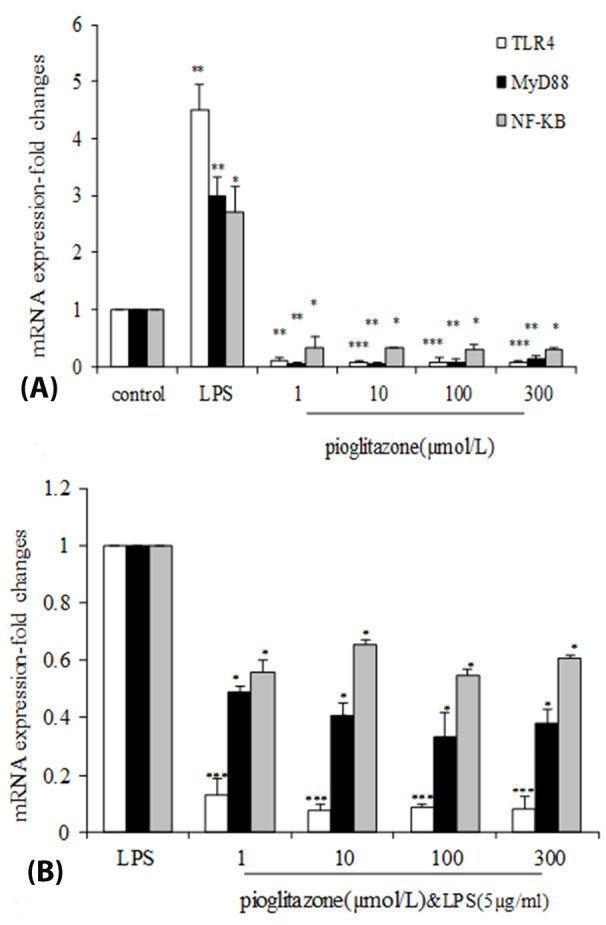
Effects of LPS-EB Ultrapure and pioglitazone treatment on expression of *Tlr4*, *Myd88* and *Nf- κ b*. (A) B16F10 cells were incubated with pioglitazone (1, 10, 100, 300 µM) or LPS (5 µg/mL)for 24 h. (B) B16F10 cells were pre-incubated for 1 h with our without pioglitazone (1, 10, 100, 300 µM) then continuing the cells incubation with LPS (5 µg/mL) for 24 h. mRNA expression was measured using quantitative real-time PCR. * *P* <0.05; **P <0.01; *** *P* <0.001 compared with negative control One representative experiment of three is depicted. Each graph has been represented as Mean ± SEM.

As shown in Figure 2A, pioglitazone treatment for 24 hours decreased the *Tlr-4*,*Myd-88* and *Nf-kB* mRNA expression in B16F10 cells by all doses. Incubation of B16F10 cells with different concentration of pioglitazone (1, 10, 100, 300 µmol/L) resulted in a dose-dependent inhibition of mRNA expression levels of *Tlr - 4* (1 (*P*  < 0.01), 10, 100, 300 µmol/L (*P*  < 0.001)).

Treatment with all doses of pioglitazone significantly reduced the expression of *Myd-88* (*P*  < 0.01),*Nf-kb* mRNA (*P*  < 0.05) compared with the control group.

Pretreatment with pioglitazone (1, 10, 100, 300 µmol/L) before LPS(5 µg/mL) (PIO+LPS) significantly decreased the induced*Tlr4* mRNA level by LPS (*P*  < 0.001) (Figure 2B). As shown in Figure 2B, after stimulation of *Myd-88* mRNA expression by LPS, pioglitazone significantly inhibited its expression (*P*  < 0.01). We also observed decreased *Nf-kb* mRNA expression in pioglitazone treated cells (*P*  < 0.05).

### Pioglitazone decreases TNF-α in B16F10 cell culture supernatant

To explore whether the effect of pioglitazone on TNF-α and IL-1β protein are mediated through TLR4, we further observed the change of the effects of pioglitazone in different concentration with or without LPS pretreatment of B16F10 cells.

**Figure 3:**
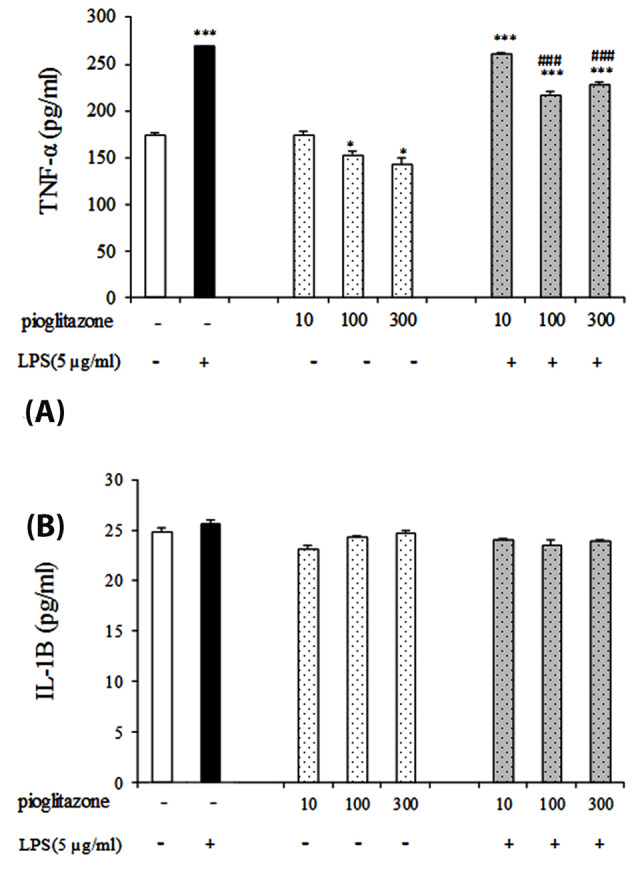
Effect of pioglitazone with or without LPS on TNF-α and IL-1β production in B16F10 cell supernatant. *P < 0.05; *** *P* < 0.001, in compare with control group and ^###^*P* < 0.001 compare LPS group. Each graph has been represented as mean ± SEM.

As shown in Figure 3A the results have shown that treatment of cells with pioglitazone alone decrease the levels of TNF-α in 100, 300 µmol/L doses compare to control group (untreated cells) (*P*  < 0.05). Moreover, TNF-α levels after treatment with pioglitazone in the combination of LPS were significantly lower than treatment with LPS alone in these doses (*P*  < 0.001). We observed no statistical changes in the IL-1β levels in pioglitazone or LPS groups after treatment (Figure 3B).

### Effect of TLR4 inhibitor on anti-inflammatory potential of pioglitazone

**Figure 4:**
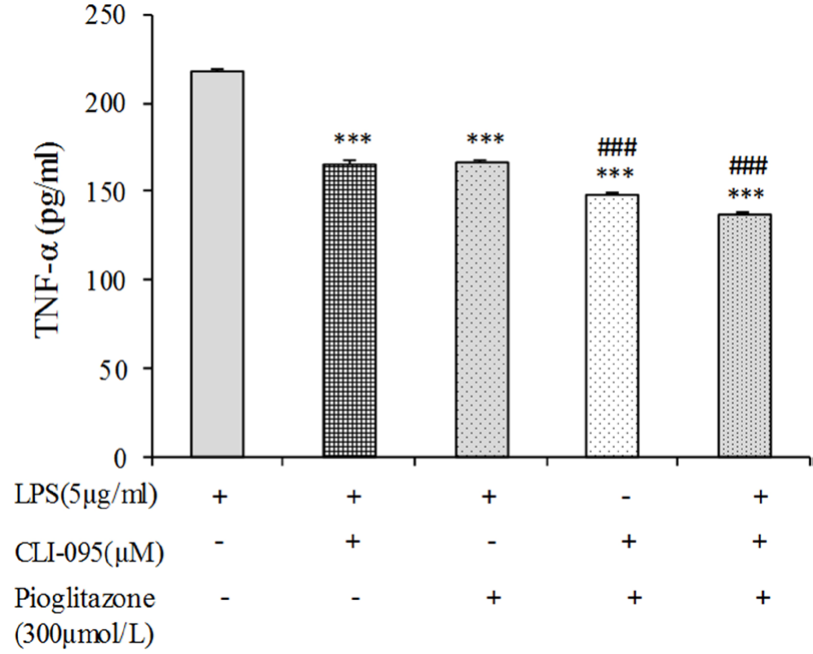
Effects of the TLR4 inhibitor, CLI-095, on the production of TNF-α induced by LPS in B16F10 cells. The cells were pretreated with pioglitazone alone or in combination with CLI-095 before LPS treatment. Following 24 h treatment, the amounts of TNF-α production was measured in the supernatants. Data are means ± SEM of three independent experiments (***P  < 0.001 compare LPS-treated cells; ^###^*P*  < 0.001 compare LPS plus pioglitazone treated cells).

As mentioned above, pioglitazone can decrease TNF-α production, and downregulate *Tlr4* expression. As shown in Figure 4, compared with the control, treatment of the cells with LPS led to TNF-α elevation, whereas the CLI-095 and pioglitazone each one alone inverted the LPS-induced effect on TNF-α in B16F10 cells. Moreover, treatment of the cells with both CLI-095 and pioglitazone synergistically reversed the effects induced by LPS in comparison with the treatment of the CLI-095 or pioglitazone alone. Considering that the TLR4 inhibitor antagonizes effects of LPS on TNF-α and pioglitazone also downregulates TLR4 expression in B16F10 cells, the modulatory effects of pioglitazone on TNF-α production in these cells is related to TLR4.

## Discussion

We have shown for the first time that pioglitazone reduced melanoma progression by suppressing TLR4 signaling pathway and inflammatory cytokines.

We observed that pioglitazone treatment alone has an anti-proliferative effect on B16F10 cells only in 300 μmol/L concentration. PPARs Play a critical role in melanoma cell proliferation and progression.^17-20^ Some studies have demonstrated the effect of PPARγ function in skin cancer and the mechanisms by which these receptors affect skin carcinogenesis, such as differentiation, proliferation, apoptosis, inflammation and angiogenesis.^21^

Several studies have shown the contradictory effects of PPAR agonists on the proliferation of melanoma cells and the mechanism underlying these growth inhibitory effects.Conﬂicting reports are based on the cell model and concentration of PPAR agonists.^22^ In the other cancers a PPARγ agonist was shown to inhibit hepatocellular carcinoma,^23^ gastric^24^ and prostate cancer cell growth.^25^ PPAR agonists exert this regulatory effect by regulation of gene expression and blocks the proto-oncogene proteins.^26^ Also They modulate NF-kB-dependent inflammatory response in innate immunity initiated by activation of TLRs.^27^

On the other hand, we observed that pioglitazone reversed the proliferative effect of LPS on B16F10 cells in all concentrations. The effect of TLR4 on cancer progression has been shown in prostate,^28^ breast,^29^ ovarian^30^ and lung cancer^31^ and it is upregulated in different cancer cells. Activation of TLR4 can inhibit melanoma cell death while TLR4 inhibition led to inhibition of their survival.^32^

Our data demonstrated that LPS treatment of B16F10 cells led to significantly increase the levels of *Tlr4*, *Myd-88* and *Nf-κb* expression in this cell, while pioglitazone reversed this effect. So we concluded that pioglitazone suppressed B16F10 cell progression by blocking the TLR4 signaling. Cross talk between different toll like receptors and PPARs have been shown in different inflammatory diseases.^14^ Previous study demonstrated that pioglitazone exert its effect against AngII-induced inflammatory effect on cardiac fibroblast cells with interferring by TLR4 signaling pathway.^33^ The other PPARγ agonist, rosiglitazone can inhibit *Tlr4* mRNA and protein expression in alveolar macrophages, which are consistent with our study.^34^ A recent study highlighted that PPARγ agonist, rosiglitazone, resisted the effect of LPS on *Tlr4*, *Myd-88* expression in esophageal cancer cells.^26^ Also, it has been shown that PPARγ 15d-PGJ2 regulates *Tlr4* mRNA and protein expression in HT-29 cells.^35^

Finally, we examine the effect of pioglitazone on TNF-α and IL-1β concentration by B16F10 cells. We observed that pioglitazone decrease the amount of TNF-α in both LPS treated and untreated cells. But it has not any effect on IL-1β. PPARγ agonists, glitazones, have established the ability to reduce inflammatory cytokine production such as TNF-α. Several studies show that thiazolidinedione have anti-tumor activity

For an assessment of this propose that pioglitazone may counteract LPS-stimulated inflammation via the blockade of TLR4, we assessed the effects of TLR4 inhibitor with or without pioglitazone on the concentration of TNF-α in B16F10. We observed that simultaneous treatment of B16F10 cells with pioglitazone and TLR4 inhibitor synergistically suppress LPS-induced levels of TNF-α. Our result has shown that the combined treatment decreased the amount of TNF-α more in comparison with the pioglitazone or TLR4 inhibitor alone treated cells. Inhibition of TLR4 binding with LPS by TLR4 inhibitor can indicate the antagonistic effect of pioglitazone against TLR4. Moreover, the negative interactions between TLR4 and PPAR-γ caused by LPS were retarded by the inhibition of the TLR4/MyD-88/NF-kB signaling pathway.

In conclusion, to our knowledge, this is the first study providing experimental evidence on effects of PPARγ activator, pioglitazone, to counter-regulate melanoma cancer by affecting on TLR4 signaling (TLR4/MyD-88/NF-kB) pathway. Further research in *in vivo* models of melanoma is needed to better understand of interaction between PPARγ and TLR4 signaling in this cancer.

## Conflict of Interest

None.

## Ethical Issues

Not applicable.

## Acknowledgments

This article is driven from a PhD thesis promoted at Isfahan University of Medical Sciences with a grant number of No. 394617. This work was financially supported by Iran National Science Foundation (INSF) [grant No. 95844116].
